# Supplementary material for: Anti-Cancer and Anti-Inflammatory Activities of Three New Chromone Derivatives from the Marine-Derived Penicillium citrinum
Source: Mar Drugs. 2021 Jul 23;19(8):408. doi: 10.3390/md19080408 (PMC8398383; doi:10.3390/md19080408)
Supplement: Supplementary file 1 [file marinedrugs-19-00408-s001.zip › marinedrugs-1307319-supplementary.pdf]

Supplementary Materials

Anti-Cancer and Anti-Inflammatory Activities of Three New Chromone Derivatives from the Marine-Derived *Penicillium citrinum*

Yi-Cheng Chu [1], Chun-Hao Chang [2], Hsiang-Ruei Liao [3], Shu-Ling Fu [1] and Jih-Jung Chen [4,5,*]

1 Institute of Traditional Medicine, School of Medicine, National Yang Ming Chiao Tung University, Taipei 112, Taiwan; chuyc.md07@nycu.edu.tw (Y.-C.C.); slfu@nycu.edu.tw (S.-L.F.)
2 Institute of Biopharmaceutical Sciences, Pharmaceutical Sciences, National Yang Ming Chiao Tung University, Taipei 112, Taiwan; changch.ps08@nycu.edu.tw
3 Graduate Institute of Natural Products, College of Medicine, Chang Gung University, Taoyuan 333, Taiwan; liaoch@mail.cgu.edu.tw
4 Department of Pharmacy, School of Pharmaceutical Sciences, National Yang Ming Chiao Tung University, Taipei 112, Taiwan
5 Department of Medical Research, China Medical University Hospital, China Medical University, Taichung 404, Taiwan
* Correspondence: jjungchen@nycu.edu.tw; Tel.: +886-2-2826-7195; Fax: +886-2-2823-2940

Contents

Figure S1. CD and ECD spectra of epiremisporine B S3

Figure S2. HRESIMS spectrum of **1** S3

Figure S3. ^{1}H NMR spectrum ($CDCl_3$, 500 MHz) of **1** S4

Figure S4. ^{13}C NMR spectrum ($CDCl_3$, 125 MHz) of **1** S4

Figure S5. ^{1}H-^{1}H COSY spectrum of **1** S5

Figure S6. ROESY spectrum of **1** S5

Figure S7. HMBC spectrum of **1** S6

Figure S8. HSQC spectrum of **1** S6

Figure S9. CD spectrum of **1** S7

Figure S10. HRESIMS spectrum of **2** S7

Figure S11. ^{1}H NMR spectrum ($CDCl_3$, 500 MHz) of **2** S8

Figure S12. ^{13}C NMR spectrum ($CDCl_3$, 125 MHz) of **2** S8

Figure S13. ^{1}H-^{1}H COSY spectrum of **2** S9

Figure S14. ROESY spectrum of **2** S9

Figure S15. Expanded ROESY spectrum of **2** S10

Figure S16. HMBC spectrum of **2** S10

Figure S17. HSQC spectrum of **2** S11

Figure S18. CD spectrum of **2** S11

Figure S19. HRESIMS spectrum of **3** S12

Figure S20. ^{1}H NMR spectrum ($CDCl_3$, 500 MHz) of **3** S12

Figure S21. ^{13}C NMR spectrum ($CDCl_3$, 125 MHz) of **3** S13

Figure S22. ^{1}H-^{1}H COSY spectrum of **3** S13

Figure S23. ROESY spectrum of **3** S14

Figure S24. HMBC spectrum of **3** S14

Figure S25. HSQC spectrum of **3** S15

Figure S26. CD spectrum of **3** S15

Table S1. The cytotoxicity data of compounds **1–3** against HT-29 cell S16

Table S2. The cytotoxicity data of compounds **1–3** against A549 cell S16

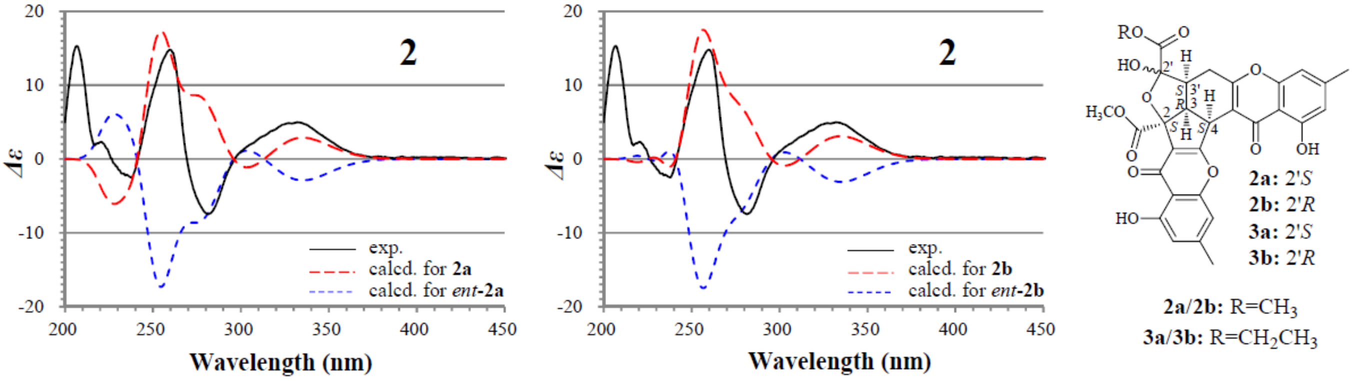

Figure S1. CD and ECD spectra of epiremisporine B.

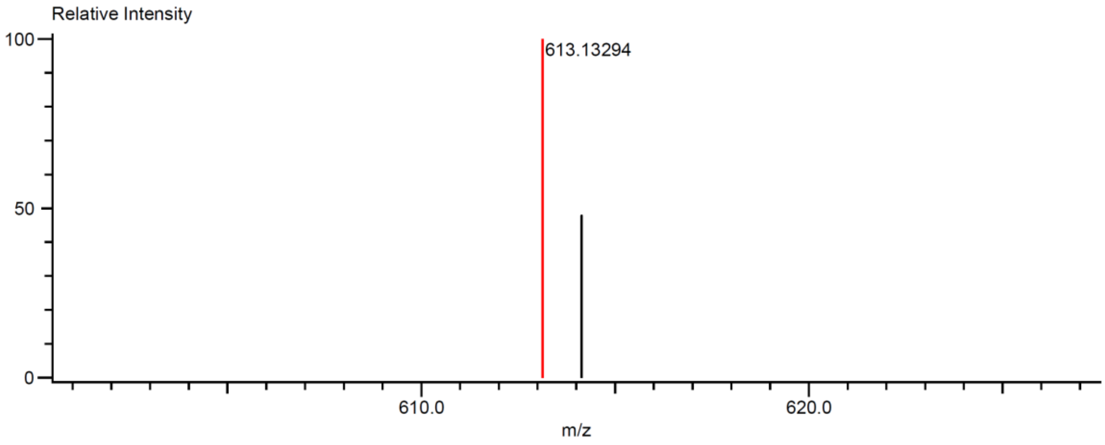

Mass	Intensity	Calc. Mass	Mass Difference [mDa]	Mass Difference [ppm]	Possible Formula
613.13294	3063.35	613.13219	0.75	1.22	$^{12}C_{31}{}^{1}H_{26}{}^{23}Na_{1}{}^{16}O_{12}$

Figure S2. HRESIMS spectrum of 1.

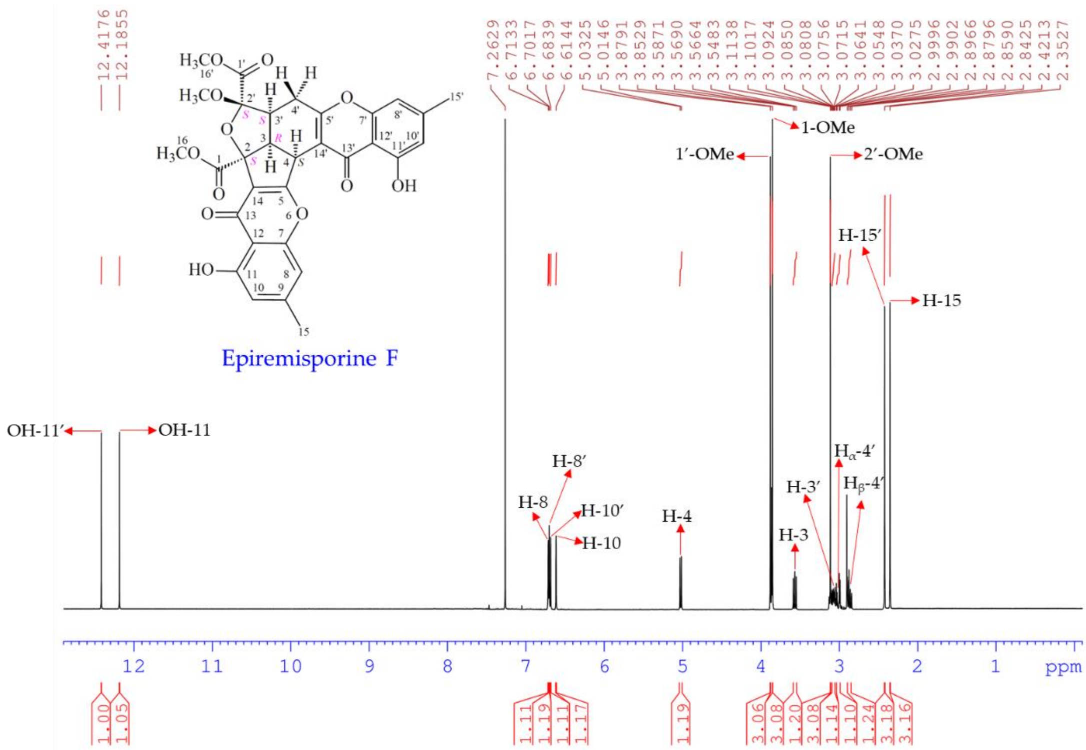

Figure S3. ^{1}H NMR spectrum ($CDCl_3$, 500 MHz) of **1**.

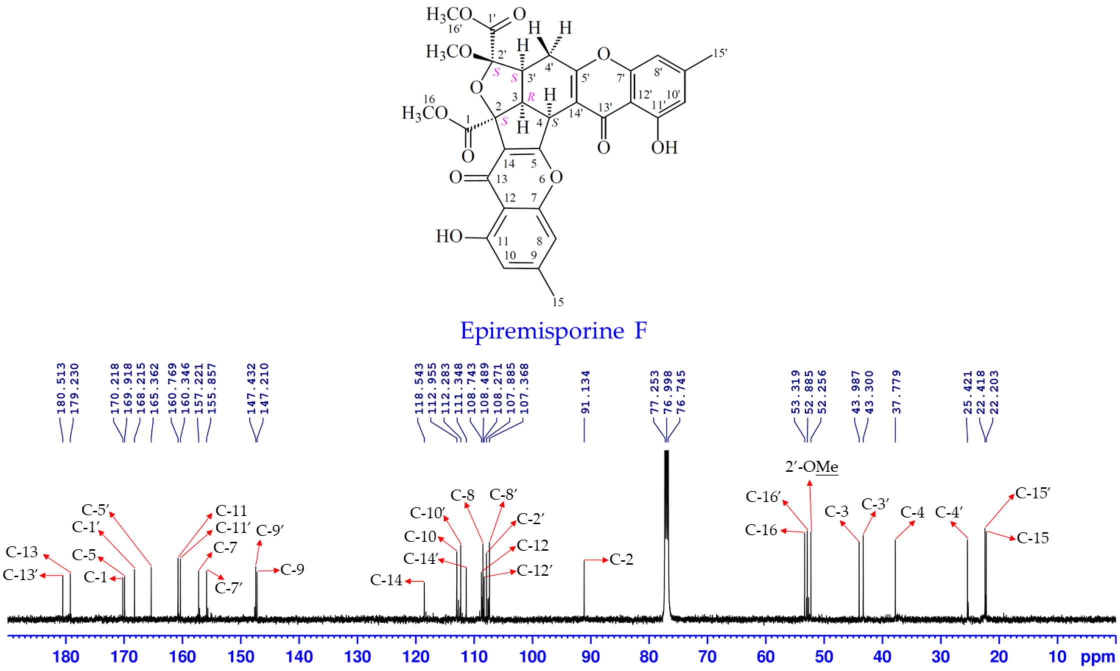

Figure S4. ^{13}C NMR ($CDCl_3$, 125 MHz) spectrum of **1**.

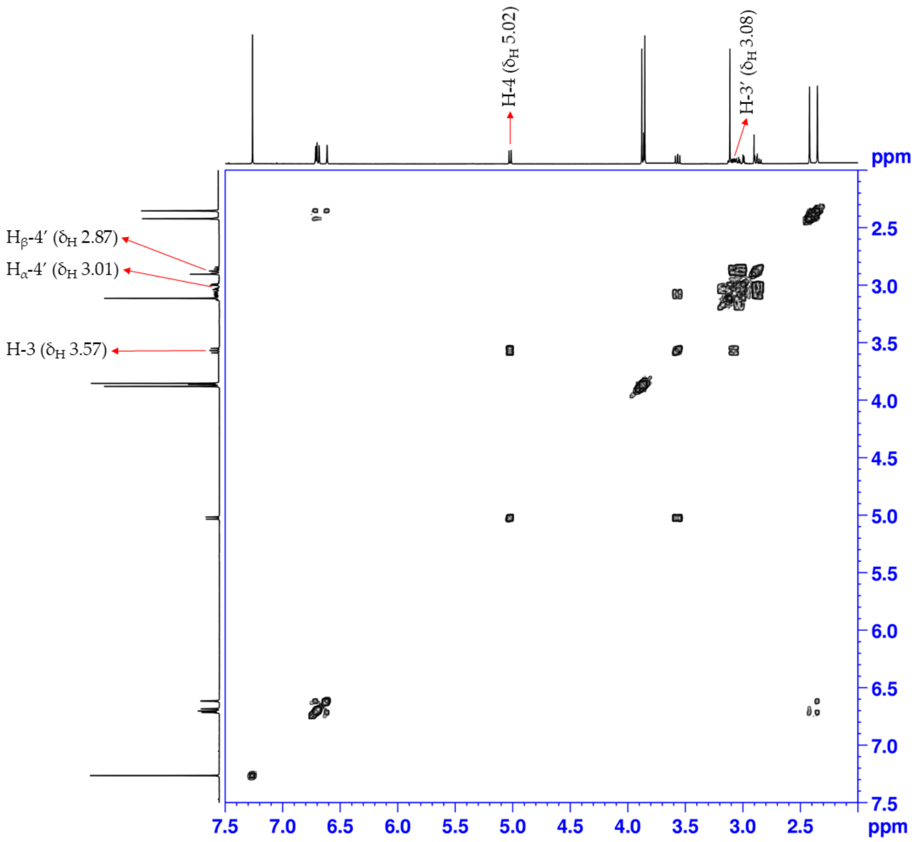

Figure S5. ^{1}H–^{1}H COSY spectrum of 1.

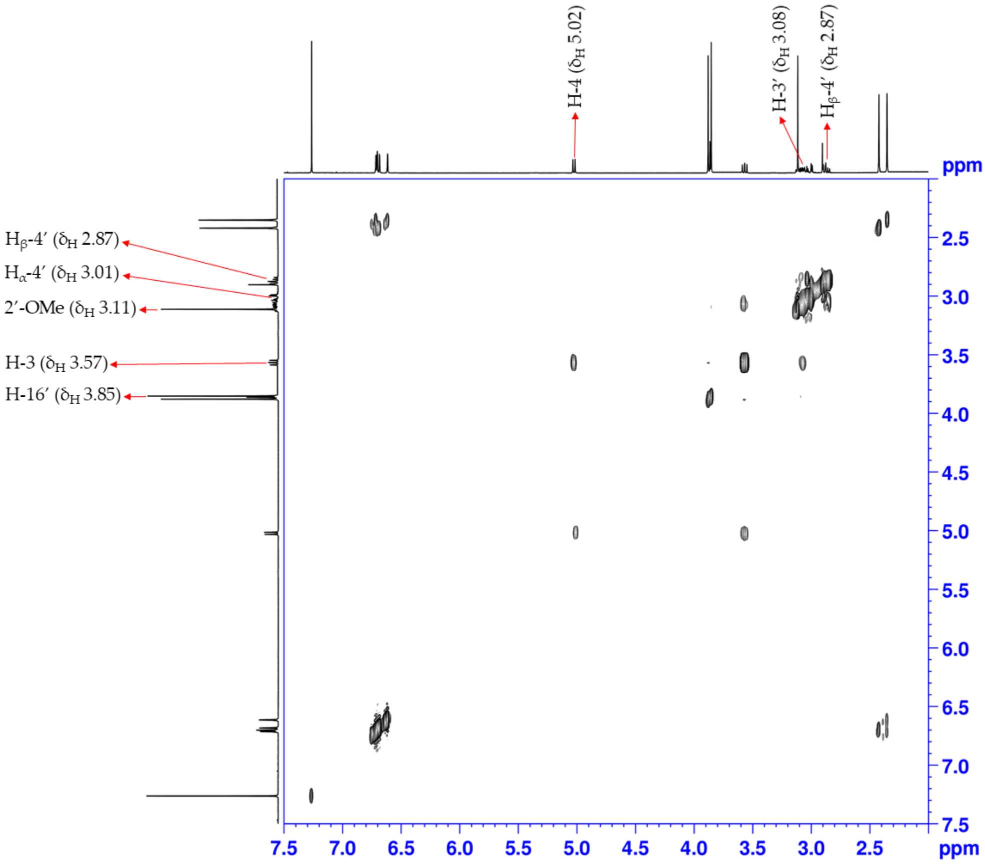

Figure S6. ROESY spectrum of 1.

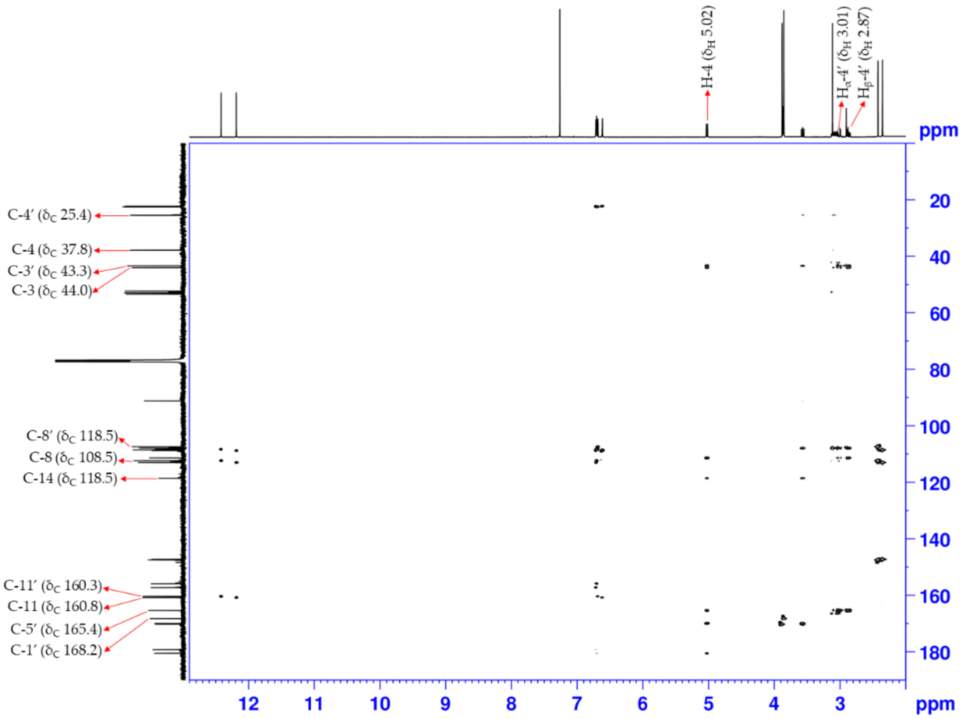

Figure S7. HMBC spectrum of 1.

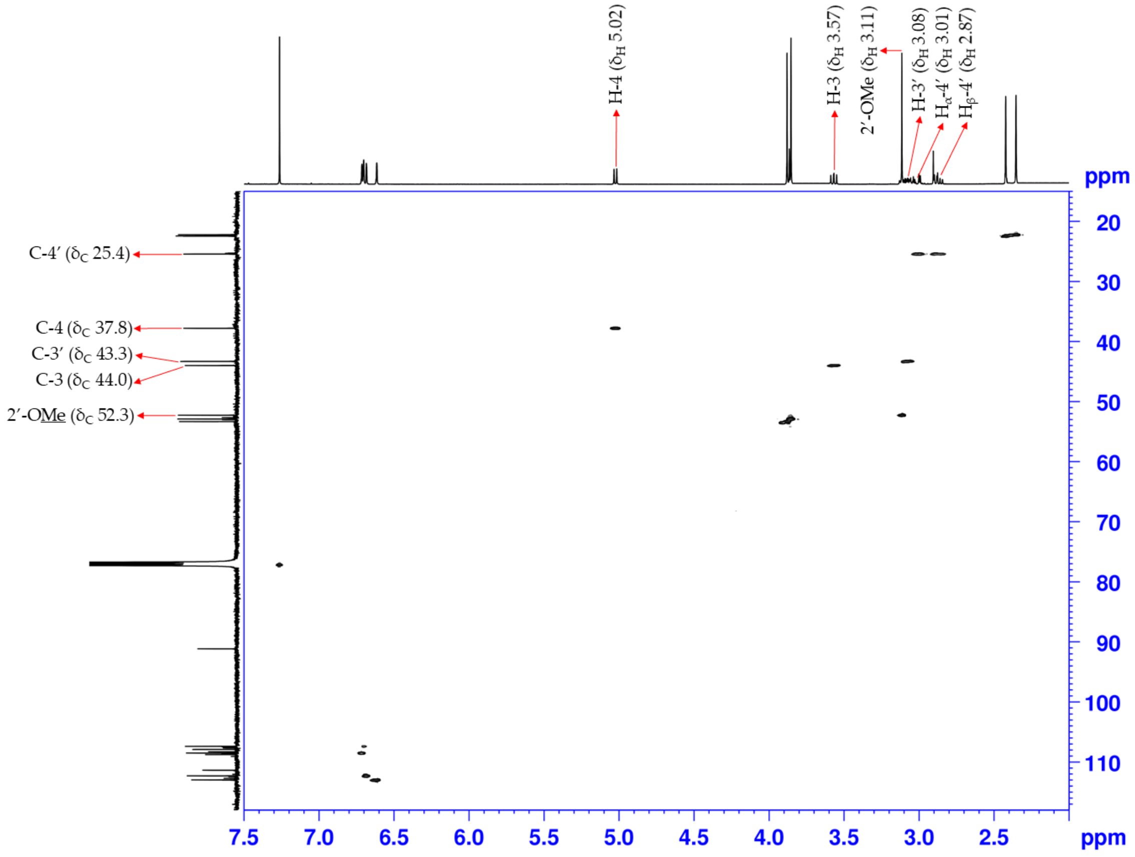

Figure S8. HSQC spectrum of 1.

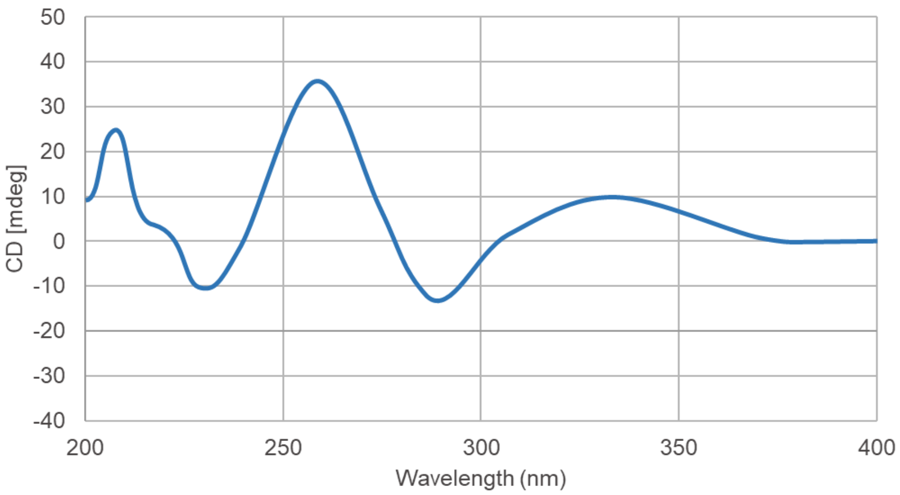

Figure S9. CD spectrum of 1.

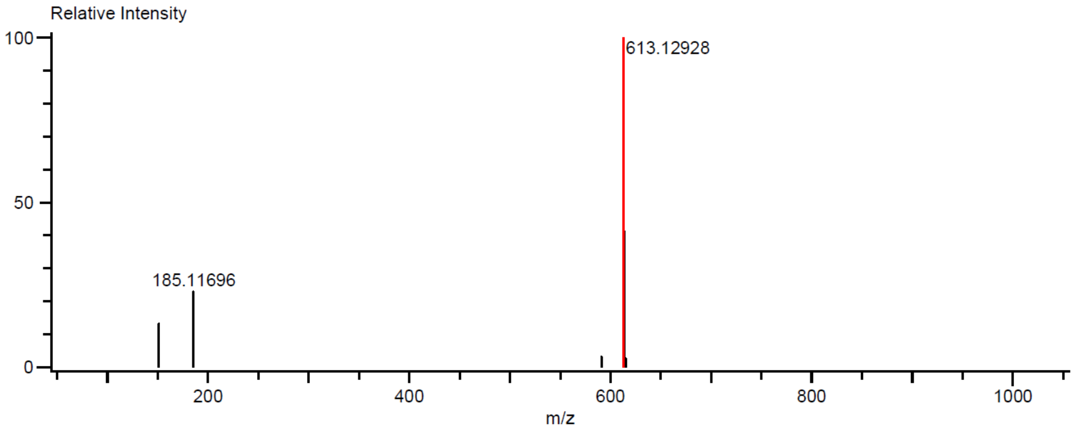

Mass	Intensity	Calc. Mass	Mass Difference [mDa]	Mass Difference [ppm]	Possible Formula
613.12928	5571.97	613.13219	-2.91	-4.75	$^{12}C_{31}{}^{1}H_{26}{}^{23}Na_{1}{}^{16}O_{12}$
		613.03829	90.99	148.42	$^{12}C_{32}{}^{1}H_{14}{}^{23}Na_{1}{}^{16}O_{12}$

Figure S10. HRESIMS spectrum of 2.

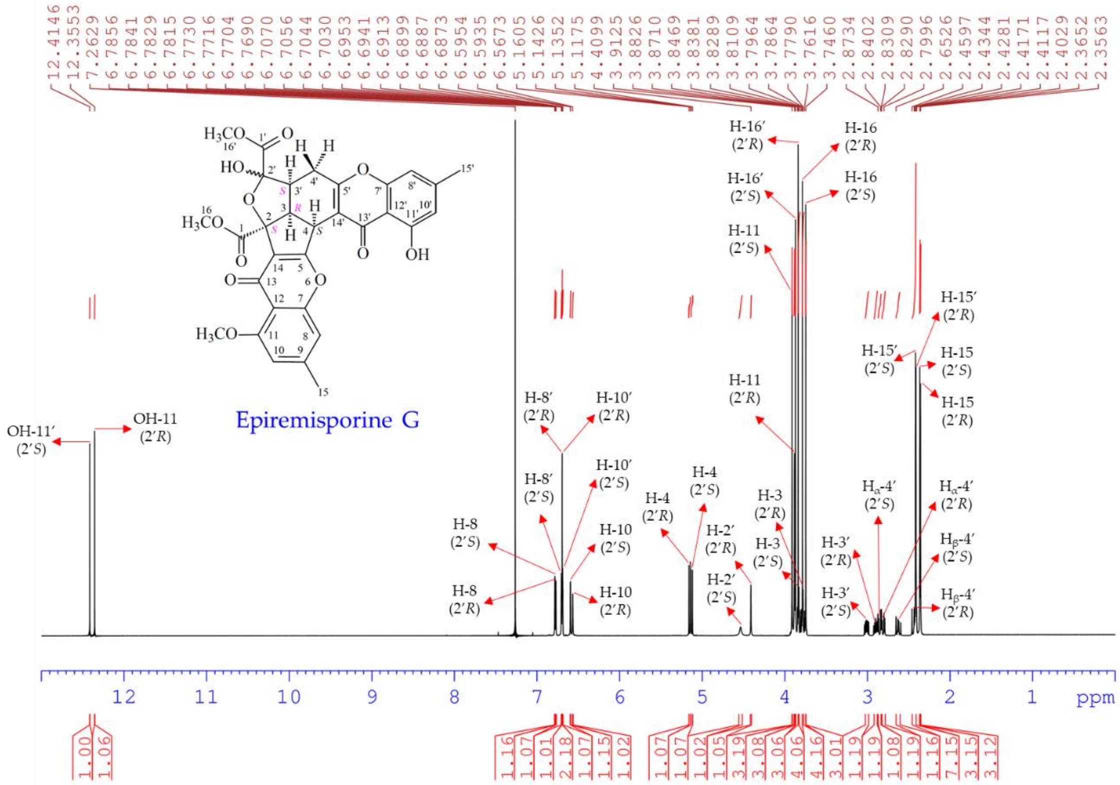

Figure S11. ^{1}H NMR spectrum ($CDCl_3$, 500 MHz) of 2.

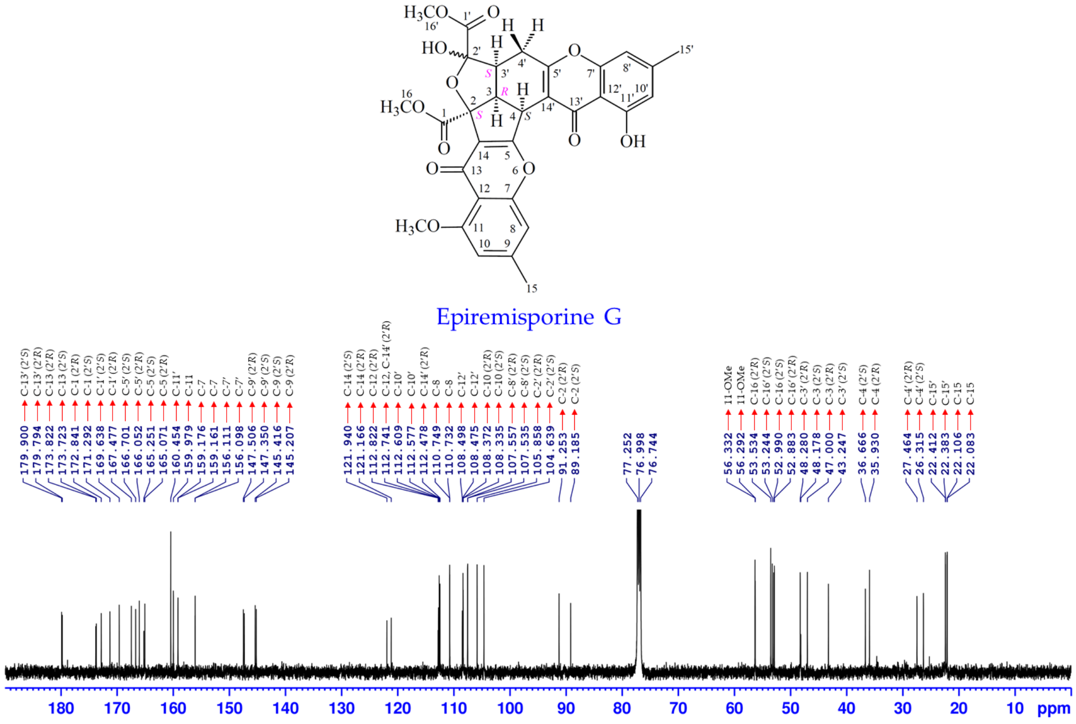

Figure S12. ^{13}C NMR spectrum ($CDCl_3$, 125 MHz) of 2.

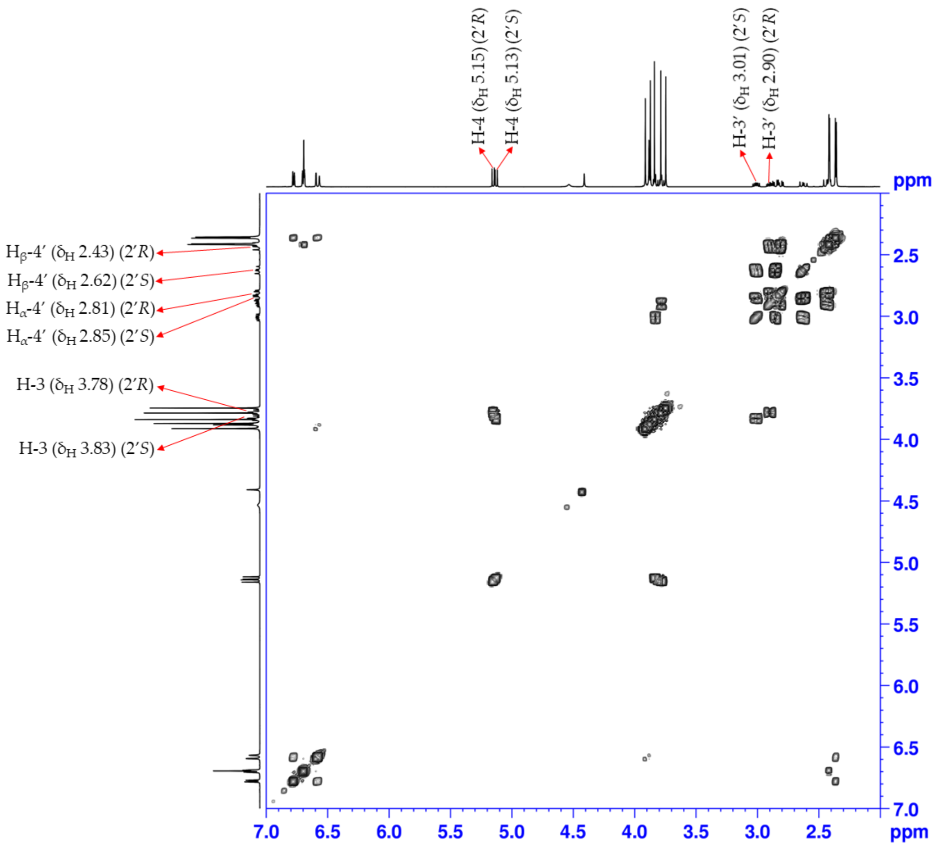

Figure S13. ^1H–^1H **COSY spectrum of 2.**

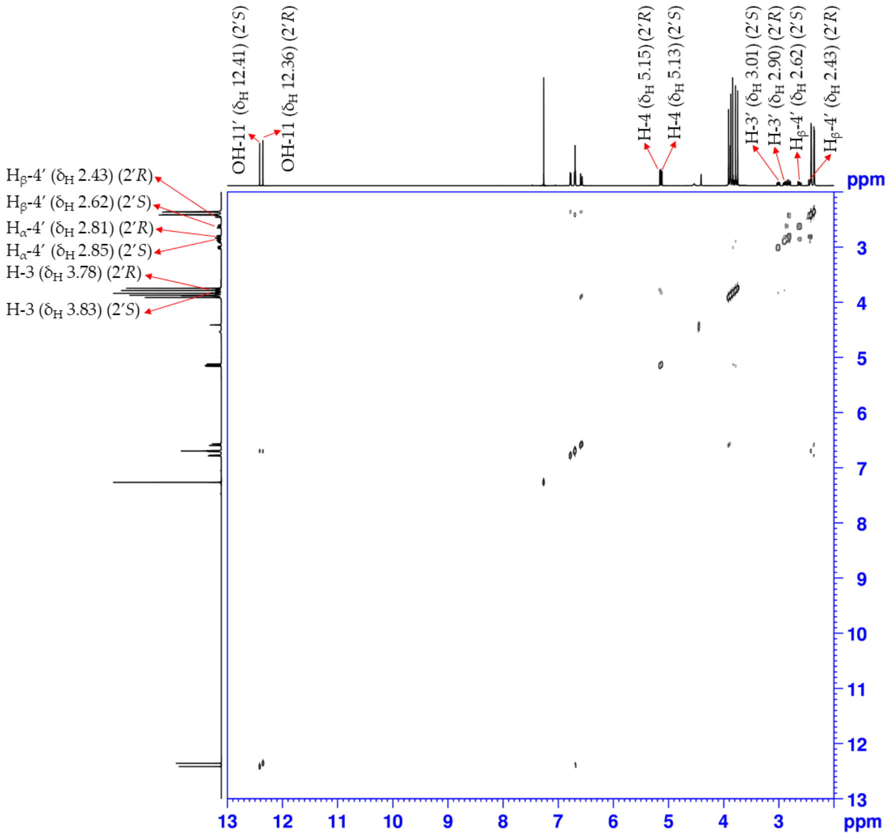

Figure S14. ROESY spectrum of 2.

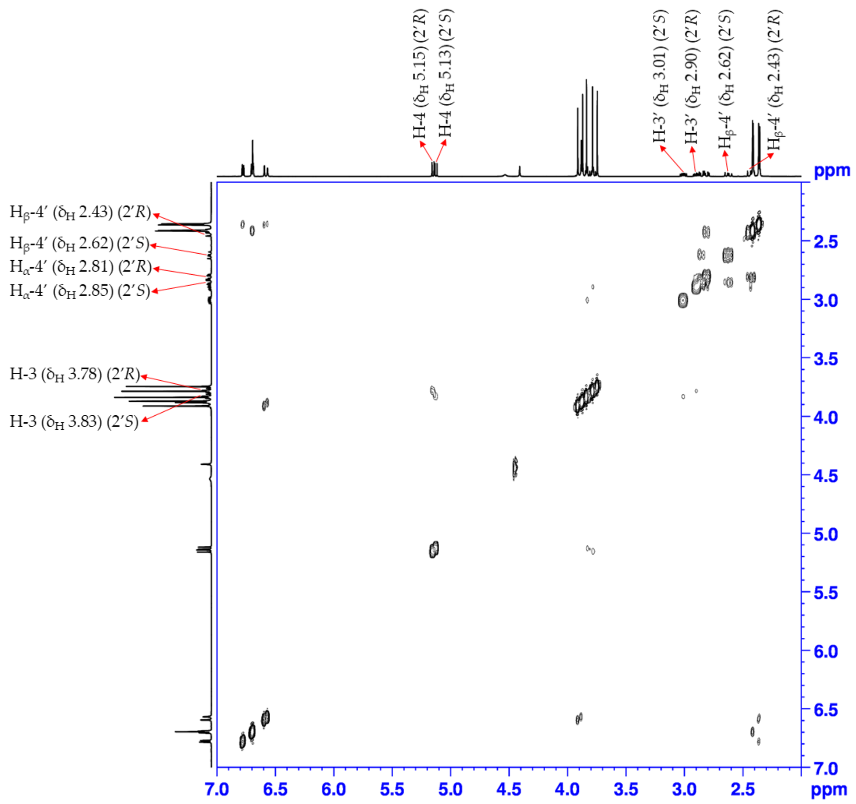

Figure S15. Expanded ROESY spectrum of 2.

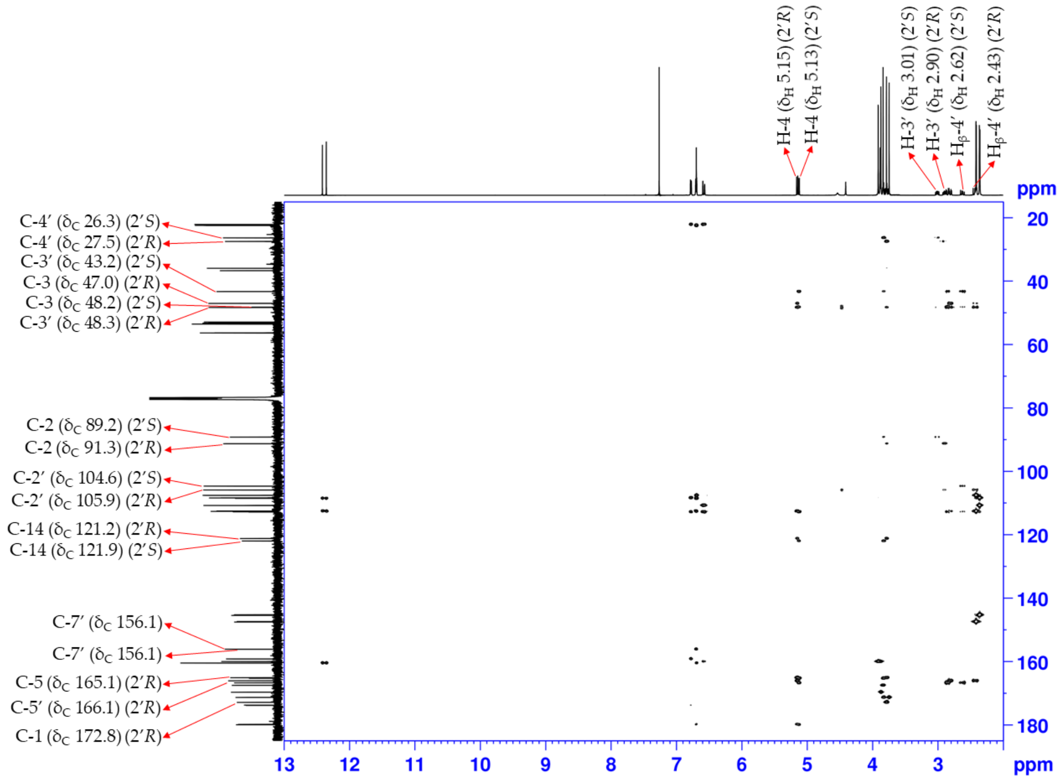

Figure S16. HMBC spectrum of 2.

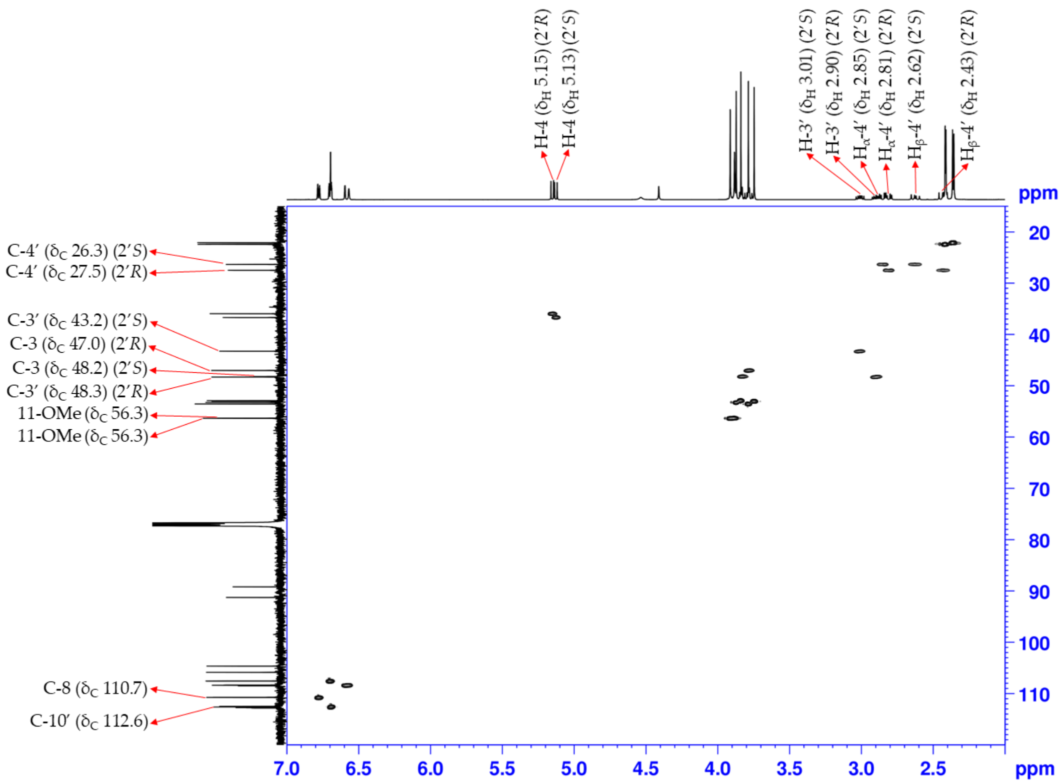

Figure S17. HSQC spectrum of 2.

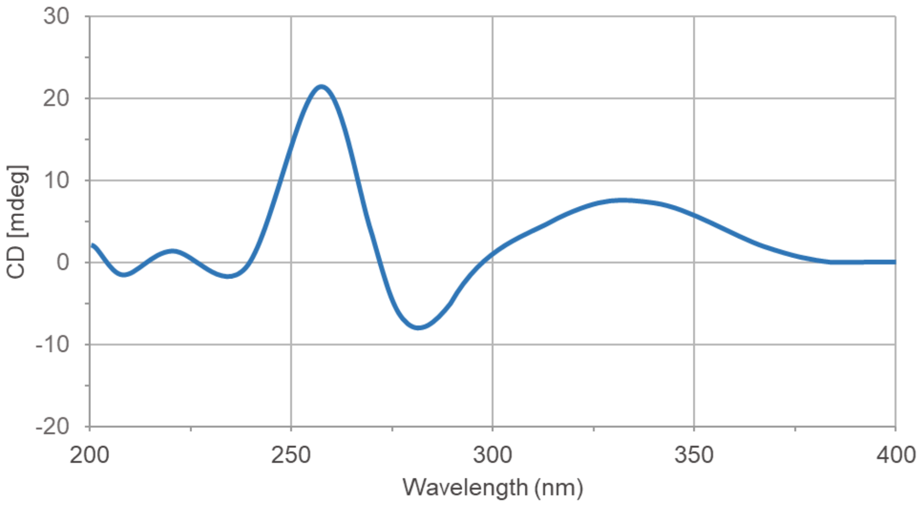

Figure S18. CD spectrum of 2.

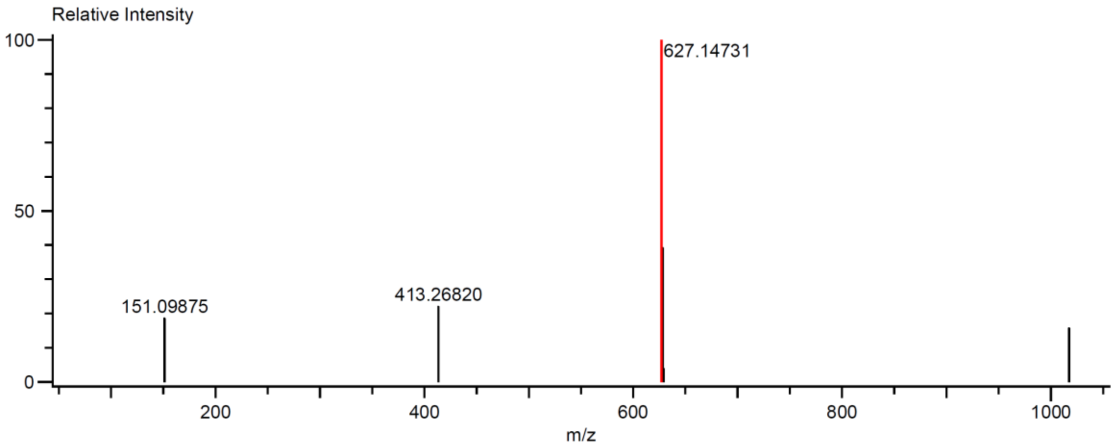

Mass	Intensity	Calc. Mass	Mass Difference [mDa]	Mass Difference [ppm]	Possible Formula
627.14731	4589.50	627.14784	-0.54	-0.86	$^{12}C_{32}{}^{1}H_{28}{}^{23}Na_{1}{}^{16}O_{12}$

Figure S19. HRESIMS spectrum of 3.

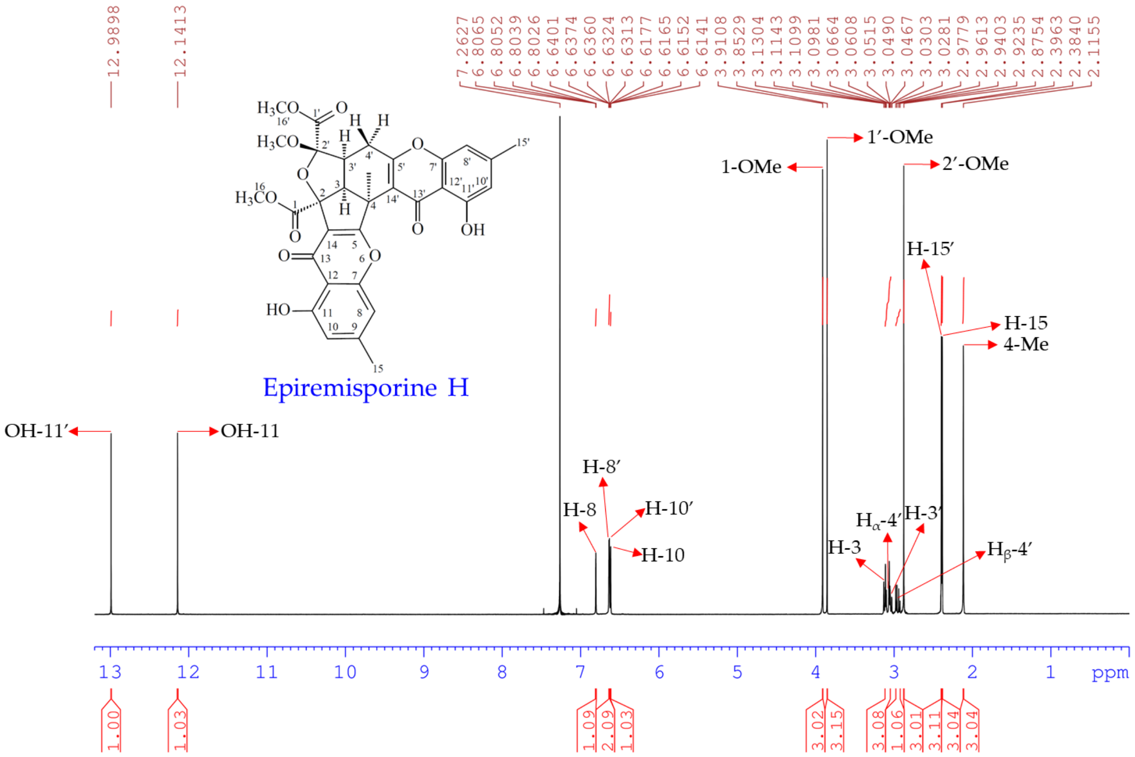

Figure S20. ^{1}H NMR spectrum ($CDCl_3$, 500 MHz) of 3.

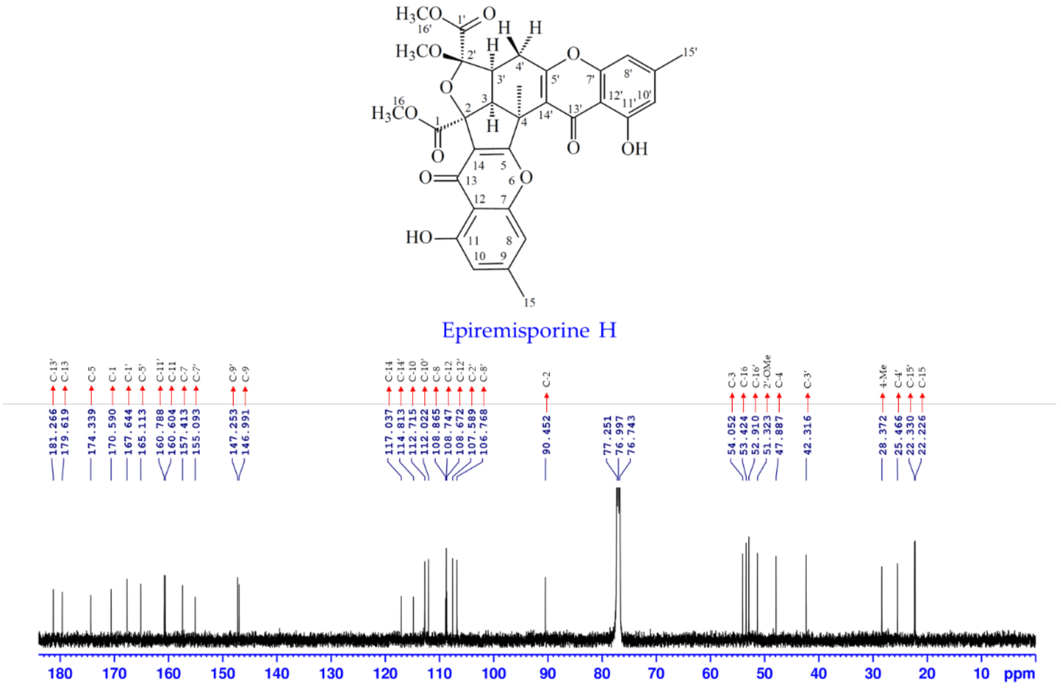

Figure S21. ^{13}C NMR spectrum of 3 ($CDCl_3$, 125 MHz) of 3.

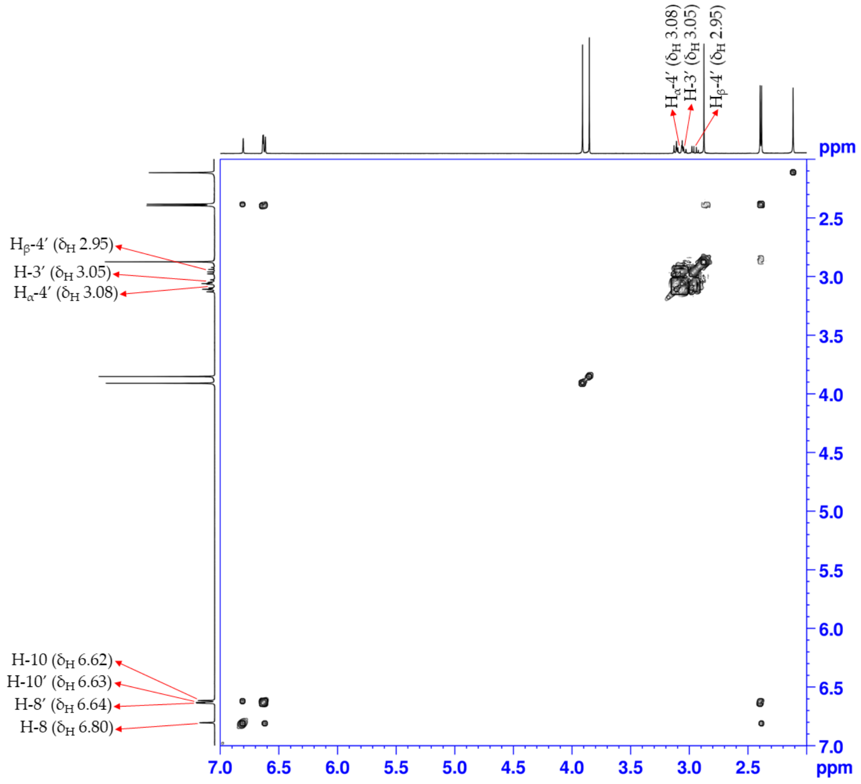

Figure S22. ^{1}H–^{1}H COSY spectrum of 3.

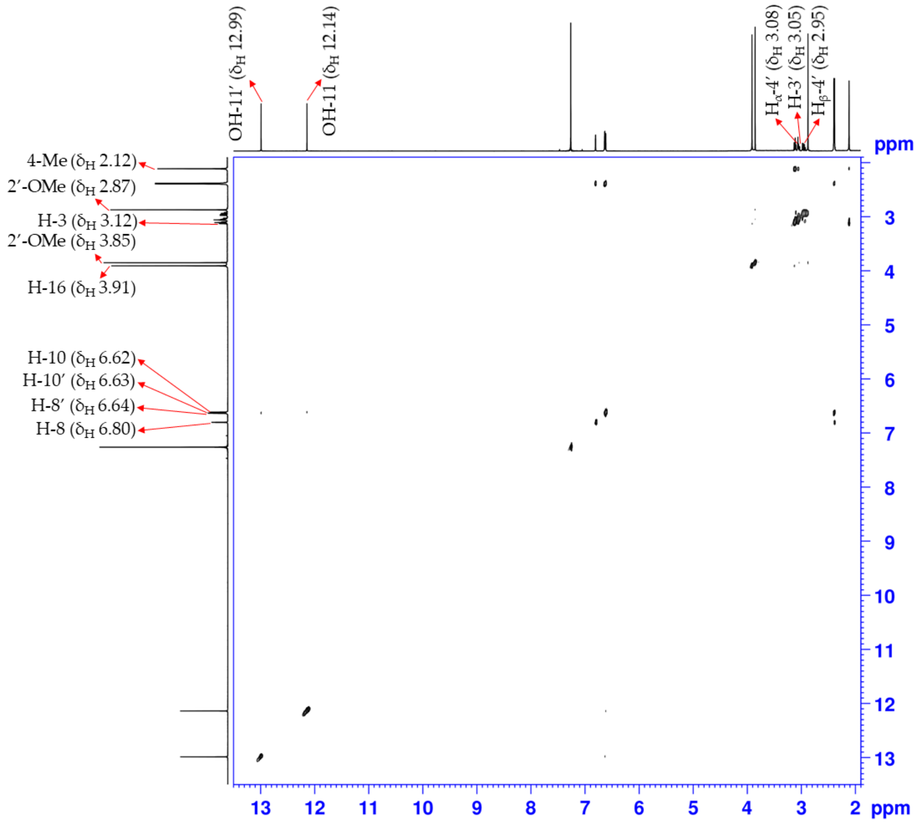

Figure S23. ROESY spectrum of 3.

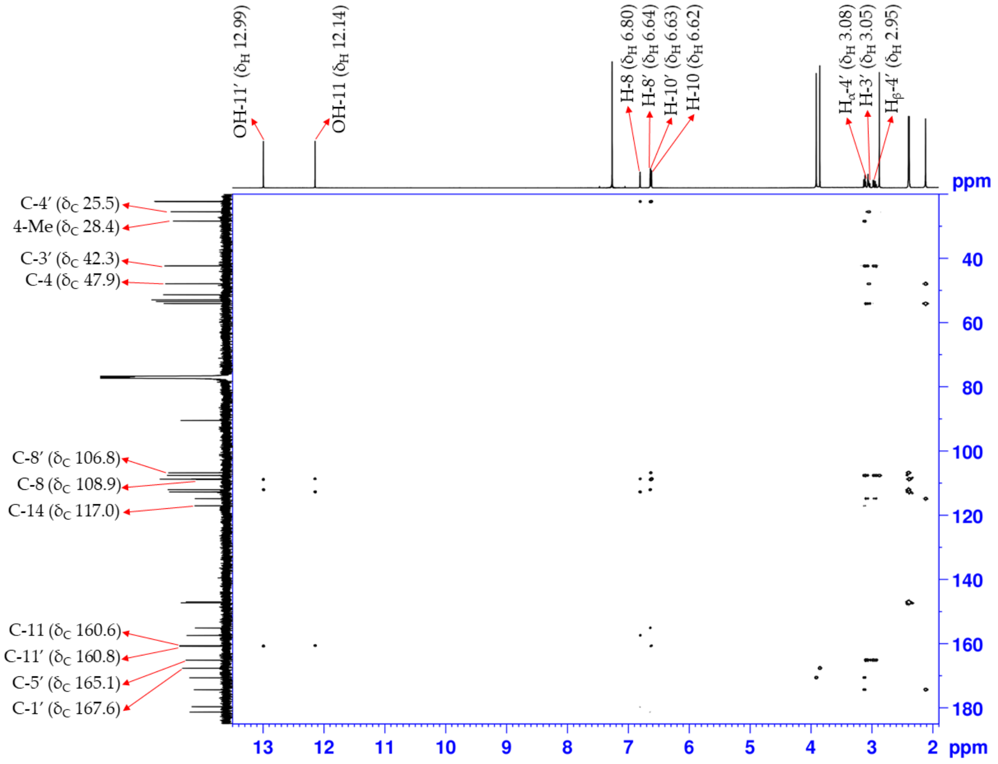

Figure S24. HMBC spectrum of 3.

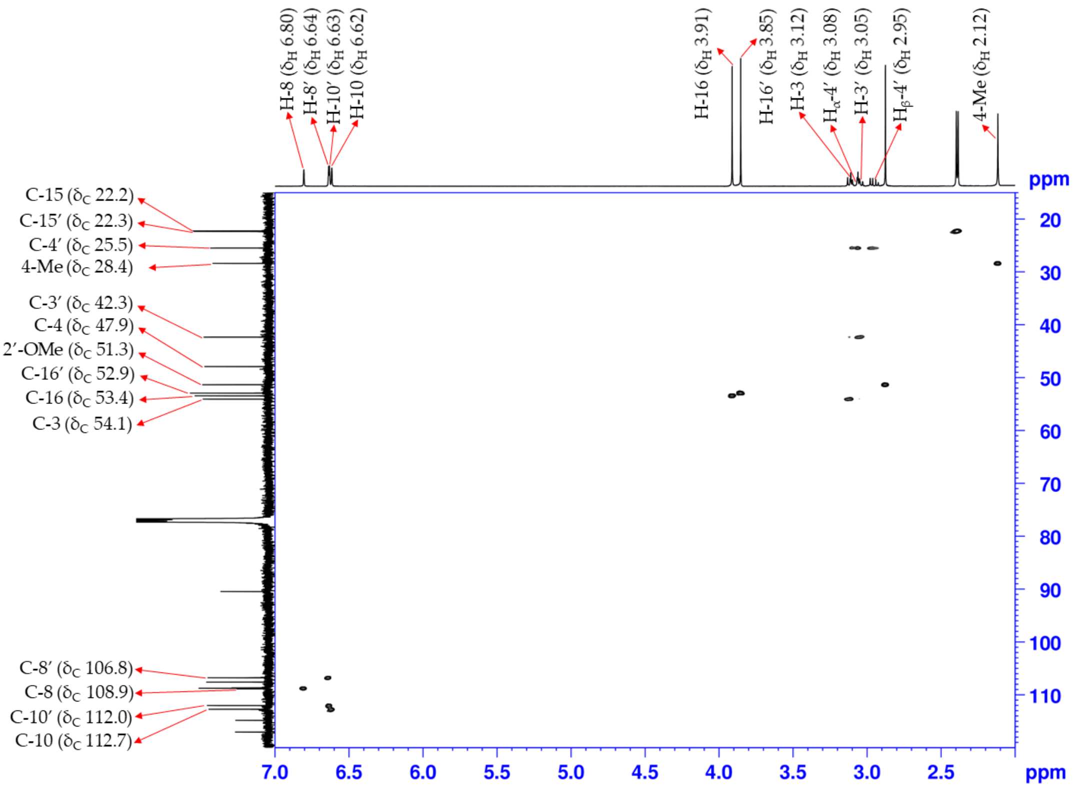

Figure S25. HSQC spectrum of 3.

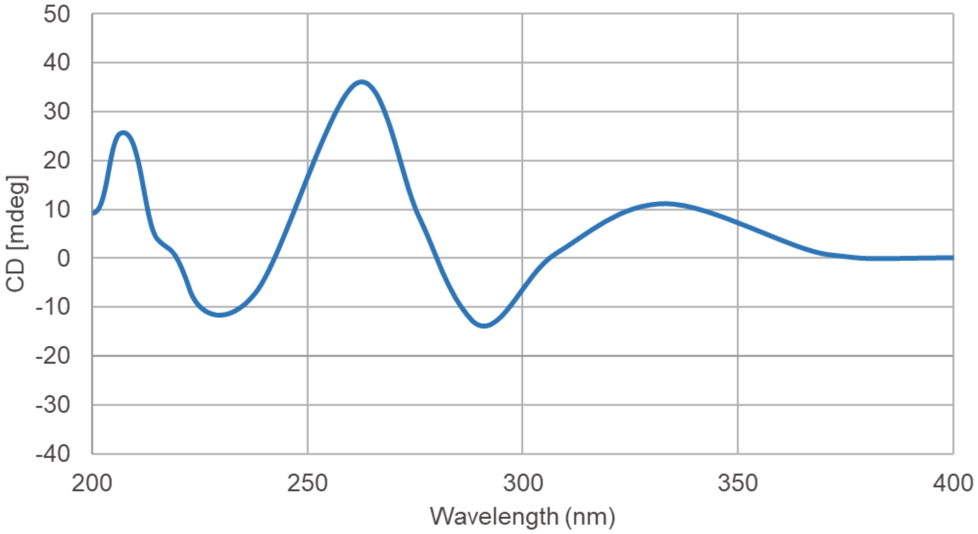

Figure S26. CD spectrum of 3.

Table S1. The cytotoxicity data of compounds **1**–**3** against HT-29 cell.

HT-29	IC_{50} (μM)		
	n=1	n=2	n=3
Ctrl	100	100	100
Epiremisporine F (**1**)	47.78	41.24	45.30
Epiremisporine G (**2**)	30.06	35.98	39.12
Epiremisporine H (**3**)	19.97	15.87	27.66
5-FU	17.88	15.32	19.20

Table S2. The cytotoxicity data of compounds **1**–**3** against A549 cell.

A549	IC_{50} (μM)		
	n=1	n=2	n=3
Ctrl	100	100	100
Epiremisporine F (**1**)	77.44	73.73	79.98
Epiremisporine G (**2**)	56.22	49.38	51.30
Epiremisporine H (**3**)	27.62	31.68	34.98
5-FU	8.20	14.91	9.58
